# Deconvolution to restore cryo-EM maps with anisotropic resolution

**DOI:** 10.21203/rs.3.rs-5976242/v1

**Published:** 2025-03-06

**Authors:** Yifan Cheng, Junrui Li, Yifei Chen, Shawn Zheng, Angus McDonald, John Sedat, David Agard

**Affiliations:** HHMI/University of California San Francisco; Howard Hughes Medical Institute; Howard Hughes Medical Institute; Chan Zuckerberg Imaging Institute; Micron School of Material Science and Engineering, Boise State University; Univ. Calif. San Francisco; University of California San Francisco

## Abstract

With technological advancements in recent years, single particle cryogenic electron microscopy (cryo-EM) has become a major methodology for structural biology. Structure determination by single particle cryo-EM is premised on randomly orientated particles embedded in thin layer of vitreous ice to resolve high-resolution structural information in all directions. Otherwise, preferentially distributed particle orientations will lead to anisotropic resolution of the structure. Here we established a deconvolution approach, named AR-Decon, to computationally improve the quality of three-dimensional maps with anisotropic resolutions reconstructed from datasets with preferred orientations. We have tested and validated the procedure with both synthetic and experimental datasets and compared its performance with alternative machine-learning based methods.

## Introduction

With the steady technological advancement over the past decade, single particle cryogenic electron microscopy (cryo-EM) has become a powerful tool for structure biology, enabling routine structure determination of many biological macromolecules at near-atomic resolution^1,2^. In theory, a three-dimensional (3D) density map is reconstructed from projection images of the macromolecules of interest with different orientations^3^. For single particle cryo-EM, the macromolecules being studied are randomly orientated in a thin layer of vitreous ice, providing projection images of the same macromolecule in all orientations^4^. Assuming a near homogenous distribution of particles orientations, the reconstructed 3D map will have an isotropic resolution. However, if particles have preferred orientations in vitreous ice, as often seen in practice, the angular distribution of projection images being used to calculate a reconstruction is uneven, with some directions under sampled, leading to anisotropic resolution of the 3D reconstruction^5,6^. In extreme cases, samples of certain orientations are entirely missing from the dataset, such as seen in electron crystallography of two-dimensional (2D) crystalline samples^7,8^, leading to information completely missing within certain angular ranges in Fourier space. This preferred orientation problem is well documented in the literature^9^. A reconstruction with severe anisotropic resolution not only distorts the density map, making it hard to interpret correctly, it also impacts accuracy of the image alignment throughout the refinement process. Since the corresponding Fourier components perpendicular to the under-sampled views are much weaker and collectively having worse signal to noise ratio (SNR), alignment of particles with such views are likely less accurate.

The root cause of preferred orientation problem in single particle cryo-EM stems from interactions of target proteins with either the air-water interface or with the supporting substrate prior to plunge freezing^6^. A classic experimental approach to deal with the preferred orientation is to tilt the specimen, which is standard in electron crystallography^8^, and it is also used in single particle cryo-EM to mitigate severe preferred orientation problem^9^. Other experimental methods include changing buffers, such as introducing detergent to change air-water interface properties, introducing substrate or modifying the expression constructs, etc., all of which require tedious trial-and-error optimizations without guarantee of success.

In addition to experimental approaches, there are also computational methods developed to restore 3D reconstructions having missing information in Fourier space. A classic computational approach of restoring the missing information in Fourier space is deconvolution^10^. Mathematically, missing information in Fourier space is treated as a point spread function (PSF) in real space convoluted with the ground truth reconstruction or image. Thus, the reverse procedure of convolution, i.e. deconvolution, can in theory be applied to restore the missing information. In general, deconvolution algorithms treat the PSF as a kernel that acts on the ground-truth volume or image. Integrating mathematical modeling, regularization and iterative processing, deconvolution approaches seek to obtain an approximation of the ground-truth volume or image. Deconvolution is extensively used in astronomy, spectroscopy, and imaging^11^. In recent years, it has also been applied to cryo-EM or cryo-STEM^12–15^.

However, most deconvolution algorithms developed for astronomy or spectroscopy^10,11^ are not only computationally complicated but also very sensitive to noise, severely compromising its applications when data is very noisy. This is particularly so for cryo-EM where the SNR of raw data is extremely poor. Entropy-regularized deconvolution (ER-Decon) is a deconvolution program that made major improvements by suppressing noise while simultaneously restoring high resolution information from widefield light microscopy images with extreme low SNR^16^. This feature makes it advantageous for dealing with low dose cryo-EM data. A few examples have now clearly demonstrated its utility when handling noisy electron tomography datasets, both cryo-EM and cryo-STEM, largely restoring missing information resulted from incomplete tilts^12–15^.

In this study, we developed a computational procedure that applies ER-Decon to improve quality of single particle cryo-EM reconstructions with anisotropic resolution caused by preferred orientations. We demonstrated that this pipeline restores the missing information in Fourier space and improves the 3D map quality by reducing its anisotropic resolution. Additionally, using a deconvolved map as a reference helps improving accuracy of angular refinement and reduces resolution anisotropy in 3D reconstructions.

## Results

### Deconvolution of cryo-EM density map

**Figure 1:**
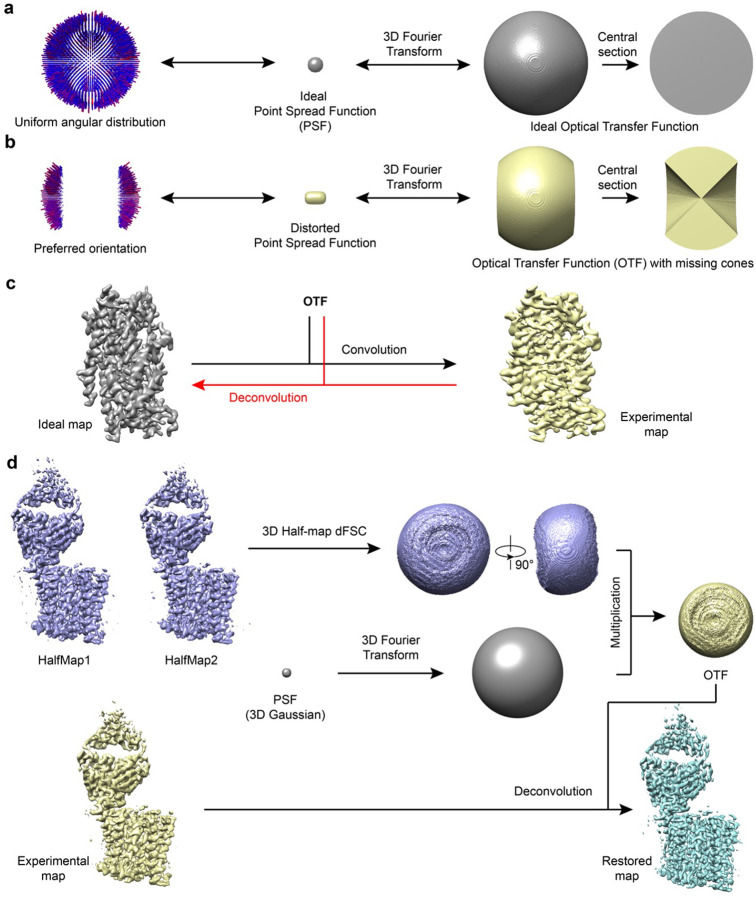
Deconvolution principle and practical pipeline **a**, Angular distribution, point spread function (PSF), and optical transfer function (OTF) for a dataset without preferred orientation. The PSF and OTF are isotropic. **b**, Angular distribution, PSF, and OTF for a dataset with preferred orientation. The PSF is elongated and the OTF shows missing cones. **c**, An experimentally obtained elongated map (yellow) can be modeled as the convolution of an ideal map (gray) with the distorted anisotropic OTF from (**b**). The black arrow indicates convolution, the red arrow indicates the deconvolution as the inverse process. **d**, The deconvolution pipeline takes two half maps and a full map from a 3D refinement job as input and outputs a deconvolved map.

Mathematically, an experimentally determined 3D cryo-EM density map can be considered as a perfect map convolved with a PSF and with added noise. The Fourier transform of an experimental map is then the product of the Fourier transform of the perfect map and the Fourier transform of the PSF, which we term as the optical transfer function (OTF). In ideal cases, where the angular distribution of all particles is uniform without any preferred orientation, the OTF would be spherical, isotropic in every direction (Fig. 1a). Assuming a simple case where images within certain angular range are completely missing, such as in random conical tilt^17^ or electron crystallographic reconstructions^8^, the OTF would be an incomplete sphere with an empty cone within which the information is missing (Fig. 1b). The PSF calculated from such an OTF with missing information is then distorted from the ideal spherical shape and the experimental density map calculated from such a dataset would be the ideal map convolved with the distorted PSF, causing elongation of the density map along the direction of missing cone (Fig. 1b and c). Computationally, the distortion of the experimental density map caused by the missing cone in the OTF, or generally by the preferred orientations, could be reversed by deconvolution, an inverse operation of the convolution^10^, which takes an experiment map and its corresponding OTF as input and iteratively optimizes an objective map to restore the map as closely as possible to the original ideal map (Fig. 1c). Regularization combined with general real space constrains, such as positivity within region of interest, are generally critical for procedures trying to minimize the consequences of missing data.

In more general and practical cases of single particle cryo-EM datasets, the angular distribution of particles is uneven with Significantly fewer images along certain orientations. The information distribution in Fourier space is then anisotropic, with poorer SNR and resolution in orientations with missing particles. This distribution of information then needs to be accurately captured in constructing an effective OTF to be used in deconvolution. To do this, we first convert the directional Fourier Shell Correlation (dFSC)^18^ calculated from two half-maps into a 3D map where the value of each voxel equals the corresponding FSC value. The OTF is then generated by multiplying this volume with the Fourier transform of a 3D spherical Gaussian PSF (essentially an overall B-factor to suppress very high frequence noise build up) and then used for deconvolving the final experimental map to restore the map with improved the resolution to as isotropic as possible (Fig. 1d).

There are many deconvolution algorithms^16,19–22^, all of which treat the PSF as a kernel that has acted upon the ground-truth image. In this study, we apply ER-Decon^16^ to partially restore the missing information in Fourier space so to improve map quality. Originally developed to restore 3D structures from widefield light microscopy images with very poor SNR, ER-Decon seeks to obtain an approximation of the ground-truth via mathematical modeling, regularization and iterative processing. Compared with other deconvolution algorithms, such as Landweber and Richardson–Lucy algorithms^23^, ER-Decon is capable of handling much lower SNRs^15^, making it more suitable for our purpose here. We name the pipeline, from generating OTF to applying ER-Decon, as AR-Decon, stands for “correcting Anisotropic Resolution by Deconvolution”.

### Deconvolution partially recovers information in the missing cone

To establish a protocol to evaluate the performance of AR-Decon, we generated a test dataset from a high quality experimental dataset collected on the ferroportin bound with a Fab^24^. The C1 reconstruction does not suffer any obvious preferred orientation (Supplementary Fig. 1a) with particles evenly distributed in Euler space (Supplementary Fig. 1b). The Fourier transform of the map shows no missing information in any direction (Supplementary Fig. 1c). The final reconstruction from this dataset has a near isotropic resolution of ~ 3Å, with minimal variation in resolution of the angular dFSC curves calculated from the two half-maps (Supplementary Fig. 1d). The OTF generated from the dFSC is thus a slightly distorted sphere (Supplementary Fig. 1e and f). From this dataset, we generated a reconstruction with anisotropic resolutions (Supplementary Fig. 1g) by purposely removing all particles with orientations more than 60° with respect to the z axis (marked), equivalent to a classic random conical tilt dataset with a missing cone of 60° aligned about the z-axis (Supplementary Fig. 1h). The Fourier transform of the reconstructed map shows a clear empty cone without information inside (Supplementary Fig. 1i). The overall resolution estimated from the FSC curve, which is equivalent to the average of dFSC along all directions, is only slightly worse than that of the original map. However, the dFSC curves in different directions vary widely, with the resolutions along the directions inside the missing cone much worse than those outside (Supplementary Fig. 1j). The 3D representation of the dFSC and the corresponding OTF have a flat disk shape (Supplementary Fig. 1k and l).

**Figure 2:**
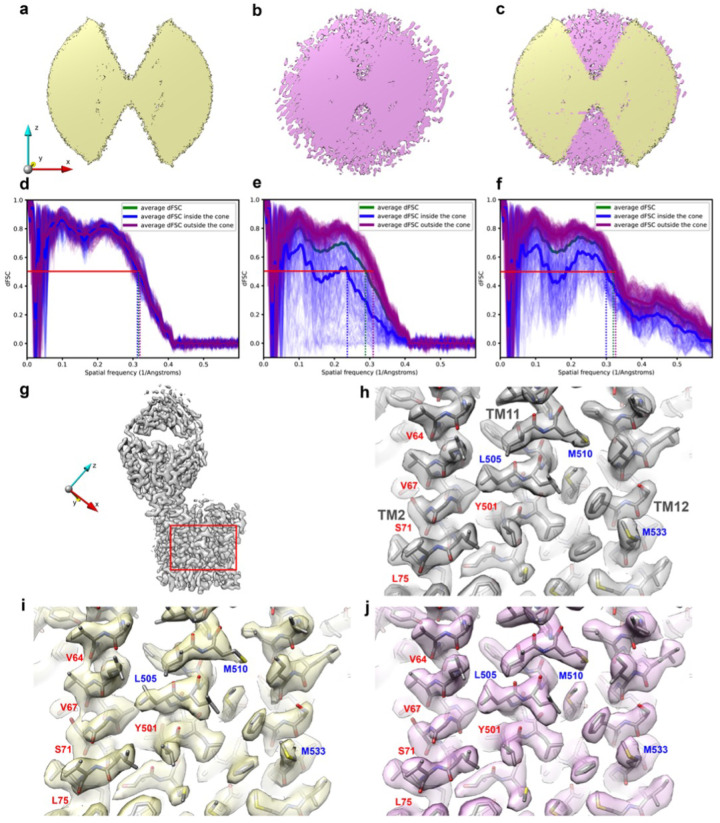
Deconvolution partially recovers information in the missing cone and improves map quality. **a**, 3D Fourier transform of the distorted map displayed at low contour level showing missing cones in z-direction. **b**, 3D Fourier transform of the map after deconvolution recovery within the missing cone in (**a**). **c**, Overlay of (**a**) and (**b**) demonstrate the missing cone filling. **d - f**, Map to model dFSC for the map without preferred orientation (**d**), the map before (**e**), after (**f**) deconvolution. The thin blue and purple lines represent the dFSC curves calculated along uniformly sampled orientations inside and outside the missing cone, respectively. The thick blue and purple lines indicate the averaged dFSC curves for these orientations, respectively. The thick green line represents the averaged dFSC across all sampled orientations. **g**, Overview of the ground truth ferroportin map without preferred orientation. The red box indicates the area shown in panels **h-j**. **h**, Zoomed view of the ground truth map in (**g**) showing protein density for three transmembrane helices TM2, TM11 and TM12. This serves as the ideal reference for comparisons. **i**, Zoomed view of the map with preferred orientation along z-axis, showing elongation of backbone density (red labels). Blue labels indicate weakened density due to preferred orientation. **j**, Both elongated backbone density and weakened density are improved after deconvolution.

Deconvolution of the distorted test map with its OTF using AR-Decon produced a “restored” map with fewer aberrations. The Fourier transform of the restored map shows that the empty cone is filled, indicating that the missing information is partially recovered (Fig. 2a–c). To evaluate the quality of information (both amplitude and phase) within the missing cone, we calculated the map to model dFSC (Fig. 2d–f). Comparisons were made based on the dFSCs of three maps (Fig. 2d–f), the original one, the one with the preferred orientation, and the restored one. Significant improvements are observed for the restored map-model correlation within the missing cone, but not to the level of the original map. The resolution difference between inside and outside the cone was Significantly reduced. By the FSC = 0.5 criterion, the average resolution estimated inside the cone was improved from 4.2 Å (Fig. 2e) to 3.38 Å (Fig. 2f) versus 3.17Å in the original map.

It is surprising that AR-Decon also slightly improved the map to model dFSC outside of the missing cone (from 3.21 Å to 3.09 Å). It is likely that deconvolution improves SNR of a target map, even without preferred orientation. To test this hypothesis, we applied ER-Decon to the original ferroportin map without preferred orientation and noticed a slight improvement of the resolution, as evaluated by the map-to-model dFSC, from ~ 3.3Å to ~ 3.1Å. This suggests that the improvement from AR-Decon is different from B-factor sharpening. In the following, we describe in details improvement of the density map after deconvolution.

Deconvolution improves the density map with missing cone, particularly reducing the elongation along the under-sampled directions. Focusing on a selected area of ferroportin (Fig. 2g), comparisons of the maps without (Fig. 2h) and with preferred orientation (Fig. 2i) show subtle but obvious differences, highlighting the influence of preferred orientations. Specifically, the map with preferred orientation shows smeared and elongated density along the *z*-axis, which is particularly obvious for helix TM2. The carbonyl oxygen densities for residues V64, V67, S71, L75, Y501 are elongated in the under-sampled directions, while the densities for L505 and M510 are weakened (Fig. 2i). Deconvolution made noticeable improvement on the map by reducing map elongation (Fig. 2j). The carbonyl oxygen density of residues V64, V67, S71, L75, Y501 are improved. The spreading out backbone density is resolved again, and the weakened densities for residues L505 and M510 were enhanced and restored to the same level as the ground truth map.

### Comparison with performance of DeepEMhancer

We also evaluate the performance of AR-Decon against DeepEMhancer^25^, which is a machine learning based method (Supplementary Fig. 2). Processing the distorted map by DeepEMhancer, with either a tightTarget or a wideTarget mask, somewhat reduced density stretching caused by preferred orientation (Supplementary Fig. 2b, c). However, detailed comparison with the deconvoluted map shows that the AR-Decon outperforms DeepEMhancer. For example, the side chain densities for L505 and M510 are less restored by DeepEMhancer than by AR-Decon. Similarly, side chain density of residue M533 on TM12 is less restored by DeepEMhancer. Overall, the density restoration by AR-Decon is in better agreement with the atomic model better than density produced by DeepEMhancer.

Another Significant improvement from deconvolution is the density of lipids that surround the protein, shown in the boxed area of ferroportin (Supplementary Fig. 2d). For a fair comparison, all maps shown in Supplementary Fig. 2e-i were at the same contour level as they were in Fig. 2. In both the ground truth map and the distorted one, the lipids densities appeared quite fragmented (Supplementary Fig. 2e, f). After deconvolution, the continuity and shape of the lipid densities are substantially improved (Supplementary Fig. 2g) and appear to be even nicer than the ground truth map at this contour level. Only when the contour level was set much lower could comparable lipid densities be seen in the ground truth and the distorted maps (Supplementary Fig. 2j, k). It was worth noting that these two maps were much noisier at lower contour level compared with deconvolved map at the higher contour level. It is likely the iterative procedure implemented in ER-Decon also improves SNR in addition to restore missing information from preferred orientation.

In contrast, lipid densities were missing in the map processed by DeepEMhancer with default tightTarget model (Supplementary Fig. 2h). Even when the contour level was set much lower, the lipids densities were not correctly resolved (Supplementary Fig. 2l). This was probably because a tightTarget model introduced a similar effect as tight masking, which removed lipids density. In the map processed with the wideTarget model, the lipids were resolved but in a discontinuous pattern (Supplementary Fig. 2i). To show comparable lipids density as the deconvolved map, the contour level also needed to be reduced Significantly (Supplementary Fig. 2m).

Overall, in the context where protein density in Fig. 2 and lipid densities in Supplementary Fig. 2e-i are displayed at the same contour level, deconvolution improved the quality of lipids densities which are close to protein density. These experiments demonstrated the ability of AR-Decon to enhance weak signal distorted by preferred orientation, while maintaining a low level of map noise.

### Deconvolution of influenza hemagglutinin (HA) trimer

**Figure 3:**
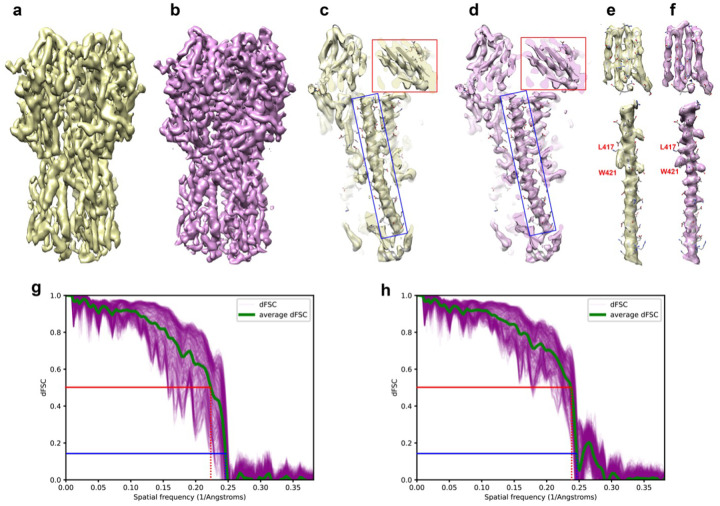
Deconvolution resolves more details in a map of influenza hemagglutinin (HA) trimer. **a**, **b**, Overview of the HA trimer map before (**a**) and after (**b**) deconvolution. Preferred orientation is along the vertical symmetric axis. **c** and **d**, Central section along symmetric axis of the HA trimer map before (**c**) and after (**d**) deconvolution. The red and blue boxes denote regions examined further in panels (**e**) and (**f**). **e** and **f**, Zoomed view of β-strands and a-helical segments in the map before (**e**) and after (**f**) deconvolution. Deconvolved map shows better separation of β-strands. Backside view of the a-helical segments shows clear helical pitches and separated side chains (red labels) after deconvolution. **g** and **h**, Map to the ground truth map (emd-21954) dFSC for the HA trimer map before (**g**) and after (**h**) deconvolution.

Single particle cryo-EM reconstruction of influenza hemagglutinin (HA) trimer (EMD-8731) reported an overall resolution of 4.2Å, with severe elongation caused by preferred orientation^9^. While β-strand and α-helices were resolved in the direction perpendicular to the preferred orientation, the map shows a strong elongation along the symmetry axis. In this direction, the map is smeared to the extent that most of the side chain density is missing (Fig. 3a). In a later study, a structure of the same protein was determined to nearly isotropic 2.9Å resolution (EMD-21954, DPB 6wxb)^26^. We now use EMD-8731 to evaluate the performance of AR-Decon on a real experimental map, and use EMD-21954 as the grand truth for comparison.

Following the same procedure, we generated an OTF from half maps dFSC plots and used it to deconvolve the EMD-8731 map. After deconvolution, the smearing effect along the preferred orientation was noticeably reduced and the helical pitches became clearly resolved (Fig. 3b). A detailed comparison of local regions in the maps prior to and after deconvolution demonstrates the improvement from the deconvolution (Fig. 3c, d). For example, in the original map, helical pitches were obscured, a tryptophan (W421) density was joined with an adjacent lysine (L417) and b-strands densities were also connected (Fig. 3c, e). In the deconvolved map, helical pitches were clearly resolved, with side chain density restored to the level sufficient for model building (Fig. 3d, f). Bulky side chains, such as tryptophan (W421) and lysine (L417), and connected b-strands were unambiguously separated (Fig. 3d, f). Using EMD-21954 as a reference map, we calculated the map-to-map dFSC for the maps before and after deconvolution (Fig. 3g, h). After deconvolution, the map-to-map dFSC exhibits reduced spreading, and the average dFSC also shows noticeable improvement.

### Deconvolved maps as references improve angular refinement

3D map refinement in single particle cryo-EM is an iterative process in which the reconstruction from the previous refinement round is used as the reference for the next iteration. In the case where particle distributions are somewhat preferred, reconstructions in the early stage of the refinement often shows obvious elongation and its Fourier transform has missing information along the direction of the preferred orientation. Taking such reconstruction as the reference for angular refinement, it would be difficult to align particles oriented outside of the preferred orientation properly, since the correlation between such images and the reference is weak. Thus, the preferred orientation problem would persist from the beginning, making it harder to recover experimentally when adding images that are outside of the preferred orientation. One idea to improve the situation is, iteratively combine deconvolution and angular refinement, using the deconvoluted map as the reference map in each cycle of the refinement, thus, to improve the alignment of images oriented within the missing orientations.

**Figure 4:**
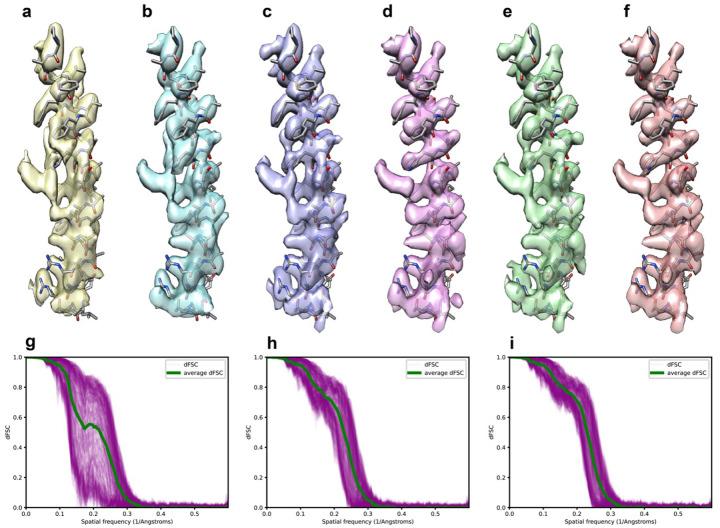
Iterative refinement and deconvolution enhance map quality and angular assignments for a preferred orientation dataset. **a**, α-helical segment from a TMEM16A map refined using a raw particle set with strong preferred orientation vertically. **b**, The same segment after deconvolution to compensate for preferred orientation. **c**, Refined map segment using the deconvolved map from (**b**) as a reference. **d**, Further deconvolution of the map in (**c**). **e** and **f**, Segments after additional round of refinement (**e**) followed by deconvolution (**f**). **g-i**, directional Fourier Shell Correlation (dFSC) curves indicate progressive improvement through each round of processing, being calculated without a mask using two half-maps from the initial (**g**), second (**h**) and final (**i**) rounds of refinement.

We tested this idea with an experimental dataset of TMEM16A, a calcium activated chloride channel (Supplementary Fig. 3). Our initial reconstruction from this sample shows clear preferred orientation (Fig. 4a). Here, we deconvolved the final reconstruction (Fig. 4b), used it as the reference and continued angular refinement (see Methods). The resultant map shows a slight improvement, as the elongation of backbone density along vertical direction reduced (Fig. 4c). We repeated the same procedure for two more rounds and noticed steady improvement of the reconstruction after each repeat (Fig. 4c–f), as shown clearly by two-half map dFSC, with much narrower spread than the original reconstruction (Fig. 4g–i). Similarly, the density, particularly side chain densities are noticeably improved (Fig. 4d, f)

### Further validation of ER-decon

In the initial process of determining a cryo-EM structure of thyrotropin receptor TSHR, a G-protein-coupled receptor (GPCR) located on thyroid follicles^27^, the sample shows a strong preferred orientation, causing a portion of the reconstruction to be uninterpretable (Supplementary Fig. 4a). In the top view of extracellular domain of TSHR (ECD-TSHR), backbones densities were smeared completely (Supplementary Fig. 4a). After deconvolution, the quality of the map in this specific region improved Significantly, resulting in separated main chain backbones densities (Supplementary Fig. 4b). Later, by experimentally improving the sample preparation and collecting a new dataset with improved angular distribution, the preferred orientation problem was partially resolved experimentally, producing a final map that was better in this region but not ideal for model building^27^ (Supplementary Fig. 4c). We then deconvoluted this improved experimental map, resulting a new map with further improved quality (Supplementary Fig. 4d). Importantly, the map deconvoluted from the initial experimental map reveal the correct folding as shown in the final experimentally improved map. This exercise validates the deconvolution procedure performed by ER-Decon.

## Discussion

In this study, we established a workflow, AR-Decon, that applies the deconvolution algorithm, ER-Decon, to improve the quality of maps with anisotropic resolution caused by preferred orientation in particle distribution. In this procedure, we first derived a OTF from dFSC plots between two half-maps, followed by deconvoluting this OTF from the experimental map by using ER-Decon. We demonstrated that this procedure could restore missing information in Fourier space and reducing anisotropic resolution (Fig. 2), thus improving map quality (Figs. 2 and 3).

While the effect of deconvolution appears similar to B-factor sharpening^28,29^, in terms of revealing high-resolution features of the map, there are some major differences. Deconvolution partly restores information within the direction of missing views, thus improving the FSC. B-factor sharpening only enhances high-resolution amplitude without filling in any additional information and thus does not improve FSC, in large part because of the noise amplification that goes along with sharpening, especially in regions with weaker signal due to limited views (Fig. 2). This tends to result in noisier, more fragmented map density. By contrast, the regularizations within ER-Decon suppress noise while simultaneously enhancing signal, helping restoring missing or limited information. Further sharpening after deconvolution often generates a better map.

This capability enables deconvolution to improve weak signal, e.g., lipid density (Supplementary Fig. 2), while keeping noise at an acceptable low level. This is an advantage over the machine learning based method, DeepEMhancer. The net result is that AR-Decon is a potentially powerful tool for cryo-EM in general, and especially for structural studies of membrane proteins that are often plagued by preferred orientation problem. The contrast with other methods is even clearer when the density of interest is at the protein-membrane interface, e.g., solvent- or lipid-exposed binding sites. Importantly, AR-Decon may help resolve binding poses of a ligand or a peptide that binds to the membrane protein at or near the interface.

Using a map after deconvolution as the reference was shown to be helpful for angular refinement when certain orientations were not equally sampled (Fig. 4 and Supplementary Fig. 3). However, the refined maps were still distorted to some extent. It is worth trying to integrate deconvolution into 3D refinement process, such that in each or every few iterations of refinement, particles in the weak direction can be aligned more correctly and more particles may potentially be identified in the direction. This leads to a better 3D reconstruction for deconvolution and further produces a better deconvolved map as a reference map for angular alignment in next iteration. Ideally, the iterative refinement and deconvolution help misaligned particles be correctly aligned and finally generate a better map.

Recently, several deep learning based approaches, such as DeepEMhancer^25^, EMReady^30^ and IsoNet^31,32^, have been developed to improve quality of maps with anisotropic resolution caused by preferred orientation. Different from explicit deconvolution method such as AR-Decon/ER-Decon, deep learning-based methods rely on clever training schemes. As demonstrated in this study, neither AR-Decon nor deep learning-based methods can perfectly restore a reconstruction with anisotropic resolution. However, combining them might be a plausible practical approach to further improve map quality. We tested this idea by combining ER-Decon with EMReady^30^. Supplementary Fig. 5c showed an HA trimmer processed firstly by AR-Decon and then EMReady. Both the connectivity and high-resolution side chains in the map (Supplementary Fig. 5c, f) were better resolved compared to the map processed solely by AR-Decon (Supplementary Fig. 5a, d) or EMReady (Supplementary Fig. 5b, e). The map to the ground truth map dFSC for the map processed by AR-Decon followed by EMReady (Supplementary Fig. 5h) is Significantly better than that of the original distorted map (Fig. 3g) and the map processed solely by AR-Decon (Fig. 3h). Furthermore, it shows noticeable improvement in the 5–6 Å frequency range compared to the map processed only by EMReady (Supplementary Fig. 5g). During model building, back-bone tracing and sidechain modeling are much easier with map processed by AR-Decon followed by EMReady than with the original distorted map.

The deconvolution algorithm, entropy-regularized deconvolution, implemented in ER-Decon program was originally developed to process 3D light microscopy images having extremely low SNR^16^. Recently, the program has been applied to electron tomography reconstruction, both cryo-EM and cryo-STEM^12,15^. Here, we demonstrated its application to single particle reconstructions suffering from anisotropic resolution caused by preferred particle orientations. It is possible that the algorithm can also be applied in other part of cryo-EM data processing pipeline where deconvolution is necessary, such as for contrast transfer function (CTF) correction.

In conclusion, by developing a deconvolution pipeline with a PSF derived from half-map dFSC plots, we were able to restore the missing information in the under-sample angular space and reduce map distortion owing to preferred orientation, thereby reducing resolution anisotropy and improving map quality. This deconvolution pipeline can be used either as a postprocessing tool, or to help 3D angular refinement.

## Methods

### Description of the AR-Decon pipline

The pipeline AR-Decon contains following parts: generating optical transfer function (OTF), applying ER-Decon II^16^ to deconvolve OTF from the target map, and an optional procedure for screening of smoothness and nonlinearity parameters for optimized performance of ER-Decon.

A key step in adopting ER-Decon for deconvolving a cryo-EM 3D volume is to obtain the corresponding 3D optical transfer function (OTF). The accuracy of OTF affects deconvolution results. Since a cryo-EM density map is a 3D reconstruction of a set of particles with different orientations, the traditional way of obtaining a PSF experimentally in fluorescence microscopy is no longer valid. In AR-Decon, we construct OTF from the two half maps of the reconstruction (Fig. 1d). For simplicity, the constructed PSF is transformed into Fourier space for easy reshaping its profile to encode the information of preferred orientations.

To deconvolve any target map, AR-Decon requires two half maps as well as the final map either sharpened or unsharpened. To generate OTF of a given target map, we first generate a volume representation of directional Fourier Shell correlation (dFSC)^18^ from two half-maps (described next). We then generate a 3D gaussian function with its parameters (m,s) initialized as below:

(1) μ=0

(2) $$\sigma =\text{res}/\left(\pi \sqrt{2}\cdot \text{ps}\right)$$

where *res* is the resolution estimate given by half-map FSC at threshold 0.143 and *ps* is the pixel size of the map. Equation (2) makes FT of the gaussian distribution fall to 1/*e* of its maximum value at frequency 1/*res*, following the definition used by a command molmap in UCSF Chimera^33^. The gaussian function was then Fourier transformed and multiplied with the 3D representation of dFSC. Their product is the OTF that we will use to deconvolve the target map.

Two major parameters that influences the performance of ER-Decon are smoothness and nonlinearity. Using synthetic Ferroportin dataset, we have screened for optimized values of these two parameters (Supplementary Fig. 6). We found that the same optimized values of smoothness and nonlinearity produce best deconvolution results for different 3D maps we tested. Thus, these values are used as default in AR-Decon. Nonetheless, a script is included in AR-Decon package to perform the same screening for any target maps.

### Calculate directional Fourier shell correlation (dFSC)

The calculation of directional Fourier shell correlation^18^ took a pair of unfiltered and unsharpened half maps as input. Instead of correlating at each spherical shell in Fourier space, we calculated the correlation for conical shells in different directions. We evenly sampled 500 directions using Fibonacci approach. For each direction, all the voxels within the con centered along this direction with apex angle of 40° were involved in the calculation. Along each direction and within the cone, a Fourier shell correlation is calculated. Once all 500 dFSCs were calculated, a 3D dFSC volume is rendered by calculating weighted average for each voxel.

### Generating masks for dFSC

A spherical mask for the target map was generated following these steps. First, a proper threshold to display the map of interest was determined in UCSF Chimera^33^. Then, the boundaries of the target density in and axes were identified. Lastly, a bounding sphere was generated according to the boundaries with a soft edge. The spherical mask was applied to a full map and two half-maps. Half-map dFSC was calculated using the pair of the masked half maps and the corresponding OTF was generated too.

### Generate a synthetic dataset of ferroportin

We started from a ferroportin dataset consisted of 310k uniformly distributed particles (Supplementary Fig. 1b) which gave a 3.1 Å resolution reconstruction^24^ (Supplementary Fig. 1a). The reconstructed map does not show any symmetry. Therefore, this particles stack can be easily tailored to produce a synthetic dataset with a desired preferred orientation. Here, without loss of generality, we generated a synthetic dataset to simulate a preferred orientation in -direction.

For each particle in the 310k particle stack, we derived a direction vector from refined Euler angles and then calculate angle between its directional vector and the z-axis. If the angle was between 0 and 60 degrees, or between 120 and 180 degrees, the particle was included, otherwise, it was removed. This process generated about 178k particles, and their angular distribution was calculated using star2build.py in pyem (https://github.com/asarnow/pyem) and displayed using UCSF Chimera^33^. The angular distribution verified that the preferred orientation was indeed along -axis (Supplementary Fig. 1h).

A full map (Supplementary Fig. 1g) and two half-maps were then reconstructed from the synthetic particle stack with preferred orientation using Relion reconstruction program^34^.

### Data processing for ferroportin maps

The ground truth map was reconstructed from all 310k particles and the distorted map for testing reconstructed from the selected 178k particles was sharpened using phenix.auto_sharpen tool^29^, and the applied B-factor was −54 and −37, respectively.

The unsharpened distorted full map was taken as input and processed with deepEMhancer with the default tightTarget model and an alternative wideTarget model. The resultant maps were sharpened with phenix.auto_sharpen tool^29^, and the applied B-factor were −28 and −18, respectively.

Deconvolution was performed on the distorted full map following the pipeline shown in Fig. 1d with a spheric mask. The spheric mask was a bonding sphere of the distorted full map shown at contour level 0.02, with edge width of 20 pixels. With the spheric mask, we calculated dFSC between two half-maps reconstructed from the selected particle stack and generated the corresponding OTF. Deconvolution parameters were set with the default values. The deconvolved map was normalized using the “normalize” processor of e2proc3d.py in EMAN2^35^.

All the maps shown in Fig. 2h–j and Supplementary Fig. 2a-i were displayed at the same contour level of 8.5 using UCSF Chimera^33^.

### Tune deconvolution parameters.

Smoothing and nonlinearity are two parameters of ER-Decon II that can be tuned for better deconvolution results. The deconvolution algorithm suggested 0.5 for smoothing parameter and 10000 for nonlinearity parameter. To identify the optimum parameters, we performed grid search for these two parameters. Smoothing parameters are selected from 5e-5, 5e-4, 5e-3, 1e-2, 2e-2, 5e-2, 1e-1, 2e-1, 5e-1, 1, 2, 5, 1e1, 2e1, 5e1, 1e2, 2e2 and 5e2. And nonlinearity varies in a set of 1, 1e1, 1e2, 1e3, 1e4, 1e5, 1e6 and 1e7. With different pairs of smoothing and nonlinearity parameters, we performed deconvolution for the same ferroportin map and calculated the map to model dFSC for the deconvolved map and plotted them side by side for comparison (Supplementary Fig. 6). In the ferroportin case, the smoothing parameter 0.5 and nonlinearity parameter 10000 generated good map to model dFSC. We found this pair of parameters worked well for many cases. They are thus used as default parameters in AR-Decon. For convenience, the script of performing similar screening is included in the AR-Decon package.

### Perform deconvolution with a mask.

Applying a mask can reduce noise, which can improve the outcomes of deconvolution. However, masking in real space is equivalent to convolving 3D Fourier transform of the mask in reciprocal space. Cautious should always be taken while using a mask to calculate half-map dFSC. It’s well known that masking may introduce artifacts within the high resolution region of FSC^36^. To avoid the bias introduced by a mask in half-map dFSC, a spheric mask with a soft edge is recommended. Generally, a shaped mask of the target protein with a soft edge is appropriate in most cases.

### Deconvolve a reconstruction of influenza hemagglutinin (HA) trimer.

The HA trimer reconstruction, together with two half maps and the mask, were downloaded from EMDB under accession number 8731^9^. We calculated dFSC between two half maps with the downloaded mask and generated the corresponding OTF. Deconvolution took the post-processed map and the calculated OTF as input and the default setting was used. The deconvolved map was sharpened using the auto sharpening tool^29^ in Phenix^37^ and the nominal resolution was set at 4.2Å.

### Perform 3D refinement and deconvolution iteratively.

Iterative refinement and deconvolution were performed on the TMEM16A dataset. In the first round of iteration, the TMEM16A particle stack was refined using non-uniform refinement^38^ tool in CryoSPARC^39^ and yielded a full map and two half maps. Half-map dFSC was calculated using two half maps along with the corresponding OTF. Then the refined full map was deconvolved with the OTF and the default smoothing and nonlinearity parameters.

In the second round of iteration, the deconvolved map was low pass filtered to 6Å and served as an initial reference for the second round of non-uniform refinement, and then followed by the second round of deconvolution. Finally, the third round of processing was performed in the same way as the second one.

### Deconvolve extracellular domain of TSHR

To focus on extracellular domain of TSHR, a mask was created using mask.py in pyem [pyem citation]. For both the old map and new map, deconvolution was performed with the mask following the pipeline shown in Fig. 1. We firstly calculated half-map dFSCs for extracellular domain of TSHR and generated corresponding OTFs. After applying the mask to the full maps, deconvolution was performed only for extracellular domain using default parameters.

## Data Availability

Deconvoluted map from the original deposited map of influenza hemagglutinin (HA) trimer (EMD-8731) is available in GitHub.

## References

[1] Cheng Y. Single-particle cryo-EM—How did it get here and where will it go. Science 361, 876–880 (2018). 30166484 10.1126/science.aat4346 PMC6460916

[2] Nogales E. The development of cryo-EM into a mainstream structural biology technique. Nature Methods 13, 24–27 (2016). 27110629 10.1038/nmeth.3694 PMC4913480

[3] Cheng Y., Grigorieff N., Penczek P.A. & Walz T. A primer to single-particle cryo-electron microscopy. Cell 161, 438–449 (2015). 25910204 10.1016/j.cell.2015.03.050 PMC4409659

[4] Adrian M., Dubochet J., Lepault J. & McDowall A.W. Cryo-electron microscopy of viruses. Nature 308, 32–36 (1984). 6322001 10.1038/308032a0

[5] Glaeser R.M. How good can cryo-EM become? Nat Methods 13, 28–32 (2016). 26716559 10.1038/nmeth.3695

[6] Glaeser R.M. & Han B.-G. Opinion: hazards faced by macromolecules when confined to thin aqueous films. Biophysics Reports 3, 1–7 (2017). 28781996 10.1007/s41048-016-0026-3 PMC5516009

[7] Nieweglowska E.S. The varphiPA3 phage nucleus is enclosed by a self-assembling 2D crystalline lattice. Nat Commun 14, 927 (2023). 36807264 10.1038/s41467-023-36526-9 PMC9938867

[8] Subramaniam S., Hirai T. & Henderson R. From structure to mechanism: electron crystallographic studies of bacteriorhodopsin. Philos Trans A Math Phys Eng Sci 360, 859–74 (2002). 12804283 10.1098/rsta.2001.0971

[9] Tan Y.Z. Addressing preferred specimen orientation in single-particle cryo-EM through tilting. Nature Methods 14, 793–796 (2017). 28671674 10.1038/nmeth.4347 PMC5533649

[10] O’Haver T. Intro to Signal Processing - Deconvolution, (University of Maryland at College Park, 2007).

[11] Jansson P.A. Deconvolution of Images and Spectra, (Dover Publications, Inc., 1984).

[12] Croxford M. Entropy-regularized deconvolution of cellular cryotransmission electron tomograms. Proc Natl Acad Sci U S A 118(2021). 10.1073/pnas.2108738118 PMC8685678 34876518

[13] Kirchweger P., Mullick D., Swain P.P., Wolf S.G. & Elbaum M. Correlating cryo-super resolution radial fluctuations and dual-axis cryo-scanning transmission electron tomography to bridge the light-electron resolution gap. J Struct Biol 215, 107982 (2023). 37268154 10.1016/j.jsb.2023.107982

[14] Sedat J. A proposed unified interphase nucleus chromosome structure: Preliminary preponderance of evidence. Proc Natl Acad Sci U S A 119, e2119101119 (2022). 35749363 10.1073/pnas.2119101119 PMC9245672

[15] Waugh B. Three-dimensional deconvolution processing for STEM cryotomography. Proc Natl Acad Sci U S A 117, 27374–27380 (2020). 33077585 10.1073/pnas.2000700117 PMC7959552

[16] Arigovindan M. High-resolution restoration of 3D structures from widefield images with extreme low signal-to-noise-ratio. Proceedings of the National Academy of Sciences 110, 17344–17349 (2013). 10.1073/pnas.1315675110 PMC3808589 24106307

[17] Radermacher M., Wagenknecht T., Verschoor A. & Frank J. Three‐dimensional reconstruction from a single‐exposure, random conical tilt series applied to the 50S ribosomal subunit of Escherichia coli. Journal of Microscopy 146, 113–136 (1987). 3302267 10.1111/j.1365-2818.1987.tb01333.x

[18] Dang S. Cryo-EM structures of the TMEM16A calcium-activated chloride channel. Nature 552, 426–429 (2017). 29236684 10.1038/nature25024 PMC5750132

[19] Dey N. Richardson-Lucy algorithm with total variation regularization for 3D confocal microscope deconvolution. Microscopy Research and Technique 69, 260–266 (2006). 16586486 10.1002/jemt.20294

[20] Sage D. DeconvolutionLab2: An open-source software for deconvolution microscopy. Methods (San Diego, Calif.) 115, 28–41 (2017). 28057586 10.1016/j.ymeth.2016.12.015

[21] Sarder P. & Nehorai A. Deconvolution methods for 3-D fluorescence microscopy images. IEEE Signal Processing Magazine 23, 32–45 (2006).

[22] Vonesch C. & Unser M. A fast thresholded landweber algorithm for wavelet-regularized multidimensional deconvolution. IEEE transactions on image processing: a publication of the IEEE Signal Processing Society 17, 539–549 (2008). 18390362 10.1109/TIP.2008.917103

[23] Sage D. DeconvolutionLab2: An open-source software for deconvolution microscopy. Methods 115, 28–41 (2017). 28057586 10.1016/j.ymeth.2016.12.015

[24] Billesbølle C.B. Structure of hepcidin-bound ferroportin reveals iron homeostatic mechanisms. Nature 586, 807–811 (2020). 32814342 10.1038/s41586-020-2668-z PMC7906036

[25] Sanchez-Garcia R. DeepEMhancer: a deep learning solution for cryo-EM volume post-processing. Communications Biology 4, 874 (2021). 34267316 10.1038/s42003-021-02399-1 PMC8282847

[26] Tan Y.Z. & Rubinstein J.L. Through-grid wicking enables high-speed cryoEM specimen preparation. Acta Crystallogr D Struct Biol 76, 1092–1103 (2020). 33135680 10.1107/S2059798320012474

[27] Faust B. Autoantibody mimicry of hormone action at the thyrotropin receptor. Nature 609, 846–853 (2022). 35940205 10.1038/s41586-022-05159-1 PMC9678024

[28] Rosenthal P.B. & Henderson R. Optimal Determination of Particle Orientation, Absolute Hand, and Contrast Loss in Single-particle Electron Cryomicroscopy. Journal of Molecular Biology 333, 721–745 (2003). 14568533 10.1016/j.jmb.2003.07.013

[29] Terwilliger T.C., Sobolev O.V., Afonine P.V. & Adams P.D. Automated map sharpening by maximization of detail and connectivity. Acta Crystallographica Section D Structural Biology 74, 545–559 (2018). 29872005 10.1107/S2059798318004655 PMC6096490

[30] He J., Li T. & Huang S.-Y. Improvement of cryo-EM maps by simultaneous local and non-local deep learning. Nature Communications 14, 3217 (2023). 10.1038/s41467-023-39031-1 PMC10239474 37270635

[31] Liu Y.-T., Fan H., Hu J.J. & Zhou Z.H. Overcoming the preferred orientation problem in cryoEM with self-supervised deep-learning. (2024). 10.1038/s41592-024-02505-1 PMC12131231 39558095

[32] Liu Y.-T. Isotropic reconstruction for electron tomography with deep learning. Nature Communications 13, 6482 (2022). 10.1038/s41467-022-33957-8 PMC9617606 36309499

[33] Pettersen E.F. UCSF Chimera -- A visualization system for exploratory research and analysis. Journal of Computational Chemistry 25, 1605–1612 (2004). 15264254 10.1002/jcc.20084

[34] Zivanov J. New tools for automated high-resolution cryo-EM structure determination in RELION-3. eLife 7, e42166 (2018). 30412051 10.7554/eLife.42166 PMC6250425

[35] Tang G. EMAN2: An extensible image processing suite for electron microscopy. Journal of Structural Biology 157, 38–46 (2007). 16859925 10.1016/j.jsb.2006.05.009

[36] Penczek P.A. Resolution Measures in Molecular Electron Microscopy. in Methods in Enzymology, Vol. 482 73–100 (Elsevier, 2010). 20888958 10.1016/S0076-6879(10)82003-8 PMC3165049

[37] Liebschner D. Macromolecular structure determination using X-rays, neutrons and electrons: recent developments in *Phenix*. Acta Crystallographica Section D Structural Biology 75, 861–877 (2019). 31588918 10.1107/S2059798319011471 PMC6778852

[38] Punjani A., Zhang H. & Fleet D.J. Non-uniform refinement: adaptive regularization improves single-particle cryo-EM reconstruction. Nature Methods 17, 1214–1221 (2020). 33257830 10.1038/s41592-020-00990-8

[39] Punjani A., Rubinstein J.L., Fleet D.J. & Brubaker M.A. cryoSPARC: algorithms for rapid unsupervised cryo-EM structure determination. Nature Methods 14, 290–296 (2017). 28165473 10.1038/nmeth.4169

